# Sec23a inhibits the self-renewal of melanoma cancer stem cells via inactivation of ER-phagy

**DOI:** 10.1186/s12964-022-00827-1

**Published:** 2022-03-02

**Authors:** Zhiwei Sun, Doudou Liu, Bin Zeng, Qiting Zhao, Xiaoshuang Li, Hao Chen, Jianyu Wang, H. Rosie Xing

**Affiliations:** 1 Institute of Life Sciences, Chongqing Medical University, 1 Yi Xue Yuan Road, Yuzhong District, Chongqing, 400016 People’s Republic of China; 2 State Key Laboratory of Ultrasound Engineering in Medicine Co-Founded by Chongqing and the Ministry of Science and Technology, College of Biomedical Engineering, Chongqing Medical University, 1 Yi Xue Yuan Road, Yuzhong District, Chongqing, 400016 People’s Republic of China; 3 The Hong Kong Polytechnic University Shenzhen Research Institute, Shenzhen, China

**Keywords:** Cancer stem cells, ER-phagy, Sec23a, FAM134B, ER stress

## Abstract

**Background:**

The genesis and developments of solid tumors, analogous to the renewal of healthy tissues, are driven by a subpopulation of dedicated stem cells, known as cancer stem cells (CSCs), that exhibit long-term clonal repopulation and self-renewal capacity. CSCs may regulate tumor initiation, growth, dormancy, metastasis, recurrence and chemoresistance. While autophagy has been proposed as a regulator of the stemness of CSCs, the underlying mechanisms requires further elucidation.

**Methods:**

The CSC component in human melanoma cell lines M14 and A375 was isolated and purified by repetitive enrichments for cells that consistently display anchorage-independent spheroid growth. The stemness properties of the CSCs were confirmed in vitro by the expressions of stemness marker genes, the single-cell cloning assay and the serial spheroid formation assay. Subcutaneous tumor transplantation assay in BALB/c nude mice was performed to test the stemness properties of the CSCs in vivo*.* The autophagic activity was confirmed by the protein level of LC3 and P62, mRFP-LC3B punta and cytoplasmic accumulation of autolysosomes. The morphology of ER was detected with transmission electron microscopy.

**Results:**

In the present study, by employing stable CSC cell lines derived from human melanoma cell lines M14 and A375, we show for the first time that Sec23a inhibits the self-renewal of melanoma CSCs via inactivation of ER-phagy. Mechanistically, inhibition of Sec23a reduces ER stress and consequently FAM134B-induced ER-phagy. Furthermore, TCGA data mining and analysis show that Sec23a is a favorable diagnostic and prognostic marker for human skin cutaneous melanoma.

**Conclusion:**

This study has elucidated a new mechanism underlying the regulation of autophagy on stemness, i.e. CSCs can exploit the SEC23A/ER-stress/FAM134B/ER-phagy axis for the self-renewal. These observations provide new ideas for exploration of the regulatory network of CSC self-renewal to develop CSCs-based therapy strategies for malignant tumors.

**Video Abstract**

**Supplementary Information:**

The online version contains supplementary material available at 10.1186/s12964-022-00827-1.

## Background

Cutaneous melanoma is known for its high rate of metastasis and mortality [1–3]. The mean survival for metastatic melanoma is less than one year. The frequent sites of metastasis are lung and the brain [1, 4, 5]. Molecular mechanisms underlying melanoma metastasis awaits further elucidation. Accumulating research evidences have shown the involvement of cancer stem cells (CSCs) in metastasis [6–8]. CSCs can be isolated from the bulk cell population from a variety of cancers and have exhibited stem cell-like features such as clonal long-term repopulation and self-renewing capacity. Although the involvement of CSCs in all aspects of human cancers have been postulated [6, 9], the mechanisms governing the regulation of CSC self-renewal remain poorly defined.

Accumulating evidence indicates that autophagic activity may promote the stemness of CSCs to augment oncogenesis and metastatic progression [10–12]. We recently reported that CSCs can exploit the autophagy-p53-Zeb1 axis for self-renewal, oncogenesis, and progression in lung cancer [13]. However, mechanistic understanding underlying the regulation of CSC self-renewal by autophagy remains limited.

Autophagy is an evolutionarily conserved biological process of energy metabolism for the maintenance of homeostasis under nutrient deprivation or other stressful conditions [14–16]. Endoplasmic reticulum (ER) is a dynamic organelle that regulates protein folding, Ca2^+^ buffering, as well as lipid and carbohydrate metabolism. Protein transportation defects may cause “ER stress” due to the accumulation of unfolded or misfolded proteins in the ER lumen. ER stress can activate ER-activated autophagy (ERAA) or ER-phagy [14, 17]. FAM134B is an ER-resident receptor. Upon sensing ER-stress, FAM134B binding to the autophagy modifiers LC3 and GABARAP activates ER-phagy [18]. Whereas regulation of CSC stemness by ER-phagy has been reported [19], underlying mechanisms are elusive.

SEC23A is an important constituent of coat protein complex II (COPII) that is responsible for the transportation of secreted proteins from rough endoplasmic reticulum to Golgi apparatus [20, 21]. As a key regulator of the secretome [22, 23], published studies, including ours, have been mainly focused on the secretome function of SEC23A [24–26]. In our previous studies, we show that miR-200c augments melanoma metastasis by targeting Sec23a [25]. We further show that S100A8 transported by SEC23A inhibits melanoma metastatic colonization via autocrine activation of autophagy in extravasated tumor cells, thus have identified for the first time the link between Sec23a and autophagy [27].

In this present study, using stable CSCs derived from human melanoma cell lines M14 and A375, we show for the first time that Sec23a inhibits the self-renewal of melanoma CSCs via inactivation of ER-phagy. Mechanistically, inhibition of Sec23a reduces ER stress and consequently FAM134B-induced ER-phagy. Furthermore, TCGA data mining and analysis show that Sec23a is a favorable diagnostic and prognostic marker for human skin cutaneous melanoma (SKCM).

## Materials and methods

### Animals

All BALB/cA-nude nude mice used in the study were obtained from the core facility of Experimental Animal Centre in Chongqing Medical University. Animal studies were conducted in accordance with an approved protocol and with the institutional animal welfare guidelines of the Chongqing Medical University.

### Cell culture

M14 melanoma cell line was kindly provided by Dr. Robert Hoffman (University of California San Diego). A375 cell line was purchased from the Cell Bank of the Chinese Academy of Sciences. The M14 and A375 cells were cultured in DMEM supplemented with 1% Amphotericin B, 1% penicillin–streptomycin and 10% FBS. M14-SE and A375-SE cells were generated using the method we previously described [28], and cultured in the DMEM/F12 supplemented with 2% B27, 1% Amphotericin B and 1% penicillin–streptomycin, 20 ng/ml EGF (Beyotime) and 20 ng/ml FGF (Beyotime).

### Reverse transcription and quantitative real-time polymerase chain reaction (RT-qPCR)

Total RNAs were isolated using Trizol (Takara, Japan) and reverse-transcribed into cDNA using PrimeScript RT Master Mix (Takara, Japan). RT-qPCR was performed using SYBR Green Real-time PCR Master Mix kit (Takara, Japan) according to the manufacturer’s instructions. The following PCR condition was used on the Light Cycler: 39 cycles of 95 °C for 30 s, 95 °C for 5 s, followed by 60 °C for 30 s in a 10 µl reaction volume. Relative expression was normalized to that of GAPDH internal control. RT-qPCR primer sequences are listed in Table 1.

**Table 1:** RT-qPCR primer sequence

Gene name	Forward primers	Reverse primers
*Aldh1*	TGGACCAGTGCAGCAAATCA	CGCCATAGCAATTCACCCAC
*CD133*	CCCCGCAGGAGTGAATCTTT	GAAGGACTCGTTGCTGGTGA
*Nanog*	AGATGCCTCACACGGAGACT	TCTGGAACCAGGTCTTCACC
*Oct4*	CGAAAGAGAAAGCGAACCAG	TGAAGTGAGGGCTCCCATAG
*Sox2*	CACAACTCGGAGATCAGCAA	GTTCATGTGCGCGTAACTGT
*Sec23a*	GTATGAAAATTTCCGCCACCTT	TATGAGTCTGTGAAGGGTTGAC
*Atg5*	TGCAGATGGACAGTTGCACA	CCACTGCAGAGGTGTTTCCA
*Bip*	CAGTTGTTACTGTACCAGCCTA	CATTTAGGCCAGCAATAGTTCC
*Pdi*	CCCTGCTGGTGGAATTCTATG	GAAGAACTTGAGCGTAGGGTAC
*Grp94*	TCTGAATTGATTGGCCAGTTTG	GGGTATCGTTGTTGTGTTTTGA
*Edem*	TCTTTGGCTACGACAACTACAT	CCAATGCATCAACAAGAGTCAA
*Atf4*	ATGGATTTGAAGGAGTTCGACT	AGAGATCACAAGTGTCATCCAA
*FAM134B*	CTGAGCTCAAGAGAAAGAAGGA	TAACTGGTCTTTGATAGCTGCA
*GAPDH*	AGAAGGCTGGGGCTCATTTG	AGGGGCCATCCACAGTCTTC

### 96-Well plate single-cell cloning assay

A single-cell suspension was prepared and the concentration was adjusted to 10 cells/ml. 100 μl of cell suspensions were seeded into each well of 96-well plates. Single-cell seeding in each well was confirmed by microscopic examination and wells containing only one cell were marked. After being cultured at 37 °C with 5% CO2 for 10 days, colonies exceeding 50 cells were counted.

### Six-well plate serial spheroid formation assay

Single-cell suspensions were plated at 1000 cells/well in six-well plates. After 2 weeks in culture, clonogenic spheroids containing > 50 cells were counted under microscopy. Spheroid cultures were then collected, and single-cell suspensions were prepared for setting up the second round of the assay. The assay was repeated for three consecutive rounds.

### Subcutaneous tumor transplantation assay in BALB/c nude mice

The 1 × 10^4^ single-cell suspensions were mixed with 50 μl Matrigel Matrix (Corning) at a 1:1 ratio. Then, 100 μl of mixture was injected subcutaneously into both insides of the hind legs of BALB/c nude mice. Tumor size was measured every 2 days and tumor volume was calculated using V = (length × width^2^)/2. Mice were euthanized when tumor volume reached ~ 1000 mm^3^.

### Western blot (WB) analysis

WB was conducted as we previously reported [27]. Cells were lysed in SDS lysis buffer (Beyotime, P0013G) containing 1% protease inhibitor PMSF (Beyotime, ST506). Extracted protein concentration was determined using the BCA protein assay kit (Beyotime, P0012S) and stored at − 80 °C. 20 μg of each protein sample were separated by electrophoresis with 12% polyacrylamide gels and transferred to polyvinylidene fluoride (PVDF) membranes (Millipore, IPVH00010). After blocking, the membranes were incubated with appropriate primary antibodies and secondary antibodies. The primary antibodies for WB were purchased from: SEC23A (Cell Signaling Technology®, #8162, 1:800), LC3B (Abcam, ab192890, 1:1000), P62 (Abcam, ab207305, 1:2000), ATG5 (Proteintech, 10181-2-AP, 1:1000), TUBULIN (Proteintech, 10068-1-AP, 1:2000), FAM134B (Proteintech, 21537-1-AP, 1:1000). The secondary antibodies of anti-Mouse (SA00001-1, 1:2000) and anti-Rabbit (SA00001-2, 1:2000) were purchased from Proteintech.

### Lentivirus production

The sequence for the sh-RNAs targeting Sec23a was 5′-GGAAGCTACAAGAATGGTTGT-3′. The sequence for the sh-RNAs targeting FAM134B was 5′-GGAUCAAAUUGAGAGUGAATT-3′. Sec23a overexpression (OE) plasmid pLVX-Puro-mRuby-Sec23a (Plasmid #36158) was provided by Addgene. The lentivirus particles of shSec23a, shFAM134B and Sec23a-OE were prepared by Sangon Biotech Co. The lentivirus particles of shAtg5 LV-ATG5-RNAi (9513-1) were purchased from GENECHEM.

### Autophagy flux analysis of LC3B puncta

Autophagy flux analysis of LC3B puncta was conducted as we previously reported [27]. Adenovirus expressing mCherry-GFP-LC3B fusion protein (Ad-mCherry-GFP-LC3B, C3011) was purchased from Beyotime. Cells were plated in 6-well plates and allowed to reach 50–70% confluence at the time of Ad-mCherry-GFP-LC3B transfection. Adenoviral infection was performed according to the manufacturer’s instructions. The presence of mRFP-LC3B puncta indicated the autolysosomes in red fluorescent images.

### Transmission electron microscopy (TEM) analysis of autolysosomes

Cells were harvested and centrifuged at 1200 rpm/min for 10 min. Cell pellet was fixed with 4% glutaraldehyde and 1% osmium tetroxide. Thereafter cell pellet was dehydrated in a graded series of alcohol and acetone and followed by embedment in Epon 816 (Electron Microscopy Sciences). Ultrathin sections were cut by a Leica ultramicrotome (Leica Microsystems) and stained with uranyl acetate and lead citrate. TEM was conducted by JEM-1400 Plus transmission electron microscope (JEOL Ltd).

### Autophagy inhibitors and activator

3-MA(M9281) was purchased from Sigma. Baf-A1(S1413) and Rapamycin(S1039) were purchased from Selleck.

### TCGA database analysis

mRNA expression of Sec23a and in human cutaneous melanoma was analyzed by TCGA Research Network (http://cancergenome.nih.gov). The survival of patients with skin cutaneous melanoma was analyzed by OncoLnc (http://www.oncolnc.org).

### Statistical analysis

All data were analyzed by Student’s independent t-test of variance using GraphPad Prism software and presented as mean ± SEM. Differences were considered statistically significant when *P < 0.05, **P < 0.01 and ***P < 0.001.

## Results

### Generation and characterization of M14 derived cancer stem cell line

**Fig. 1:**
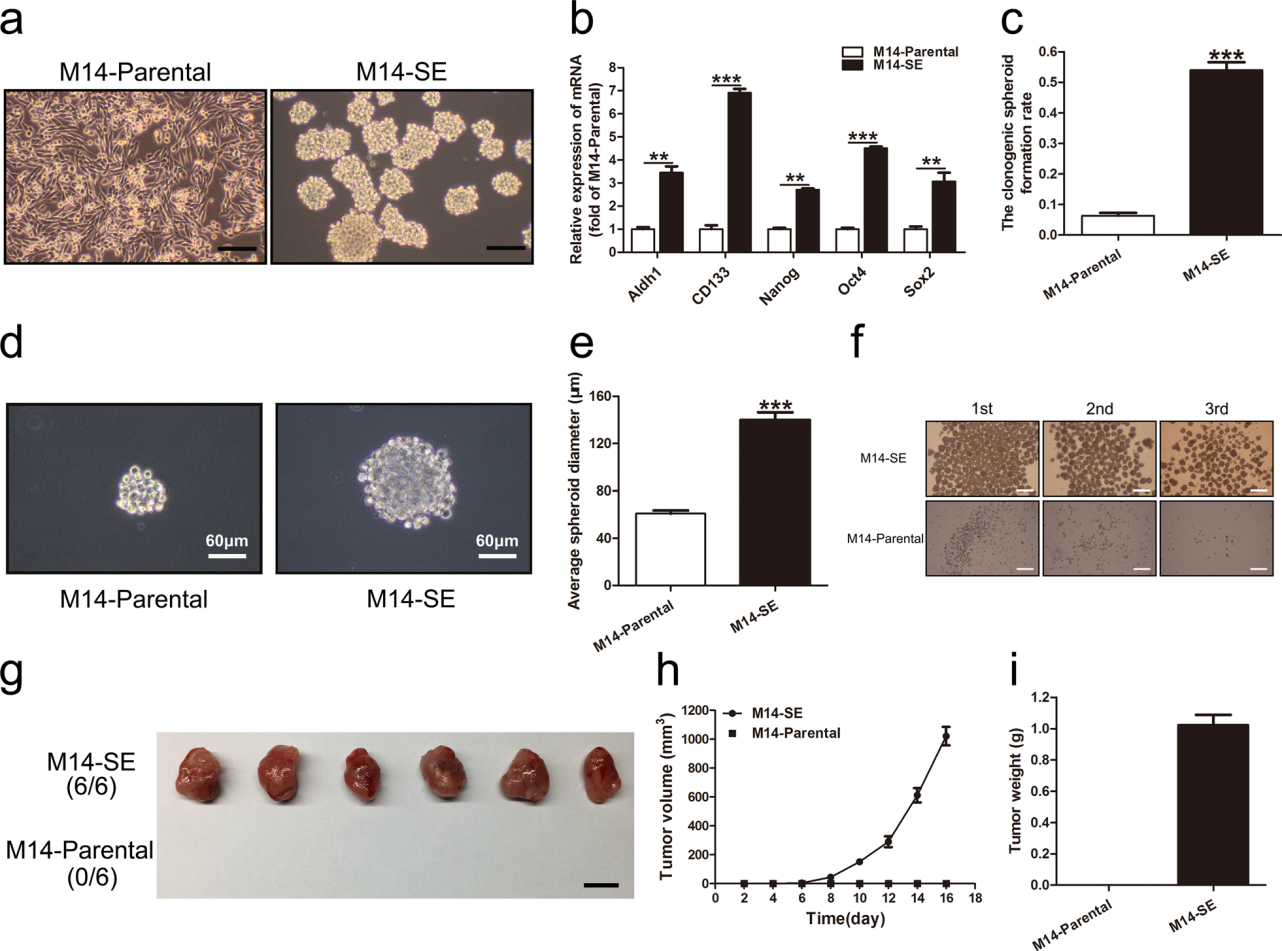
Generation and characterization of M14 derived cancer stem cell line. **a** The morphology of M14-Parental and M14-SE cells, bar = 120 µm. **b** Analysis of mRNA expression of Aldh1, CD133, Nanog, Oct4 and Sox2. The GAPDH expression was used as a reference control. **c**–**e**. Clonogenic spheroid formation rate, morphology and average spheroid diameter of M14-Parental and M14-SE cells in the single-cell cloning assay. **f** Serial spheroid formation assay of M14-Parental and M14-SE cells, 1st,2nd and 3rd means the first, second and the third round of the spheroid assay. bar = 200 µm. **g** Tumors resected from nude mice inoculated with M14-Parental and M14-SE cells, respectively, bar = 1 cm. **h** and **i**. Tumor volume and weight, **P < 0.01, ***P < 0.001

The subpopulation of CSCs in M14 cell line was established by enriching the floating spheroids in the defined stem cell media, a method we have developed and previously described [28]. The established CSCs cell line was named M14-SE and its control cell line was named M14-Parental (Fig. 1a). The enhancement of the stemness properties of the M14-SE cells was confirmed in vitro by: (1) increased expression of stemness markers including Aldh1, CD133, Nanog, Oct4 and Sox2 (Fig. 1b); enhanced self-renewal measured by (2) single-cell cloning assay (Fig. 1c–e) as well as by (3) serial spheroid formation assay (Fig. 1f). In vivo, 10^4^ cells of M14-Parental and M14-SE cells were injected subcutaneously into BALB/c nude mice. Tumors were exercised and weighed on day 16-day post tumor cell inoculation. M14-Parental cells failed to develop into tumors in nude mice while the M14-SE cells successfully grow into evenly sized tumors in all inoculated mice (Fig. 1g–i). Collectively, both in vitro and in vivo experiments had confirmed that M14-SE cells represented the CSC component of M14-parental, and were more oncogenic in comparison with the M14-Parental cells.

### Sec23a inhibits the stemness of M14-SE cells

**Fig. 2:**
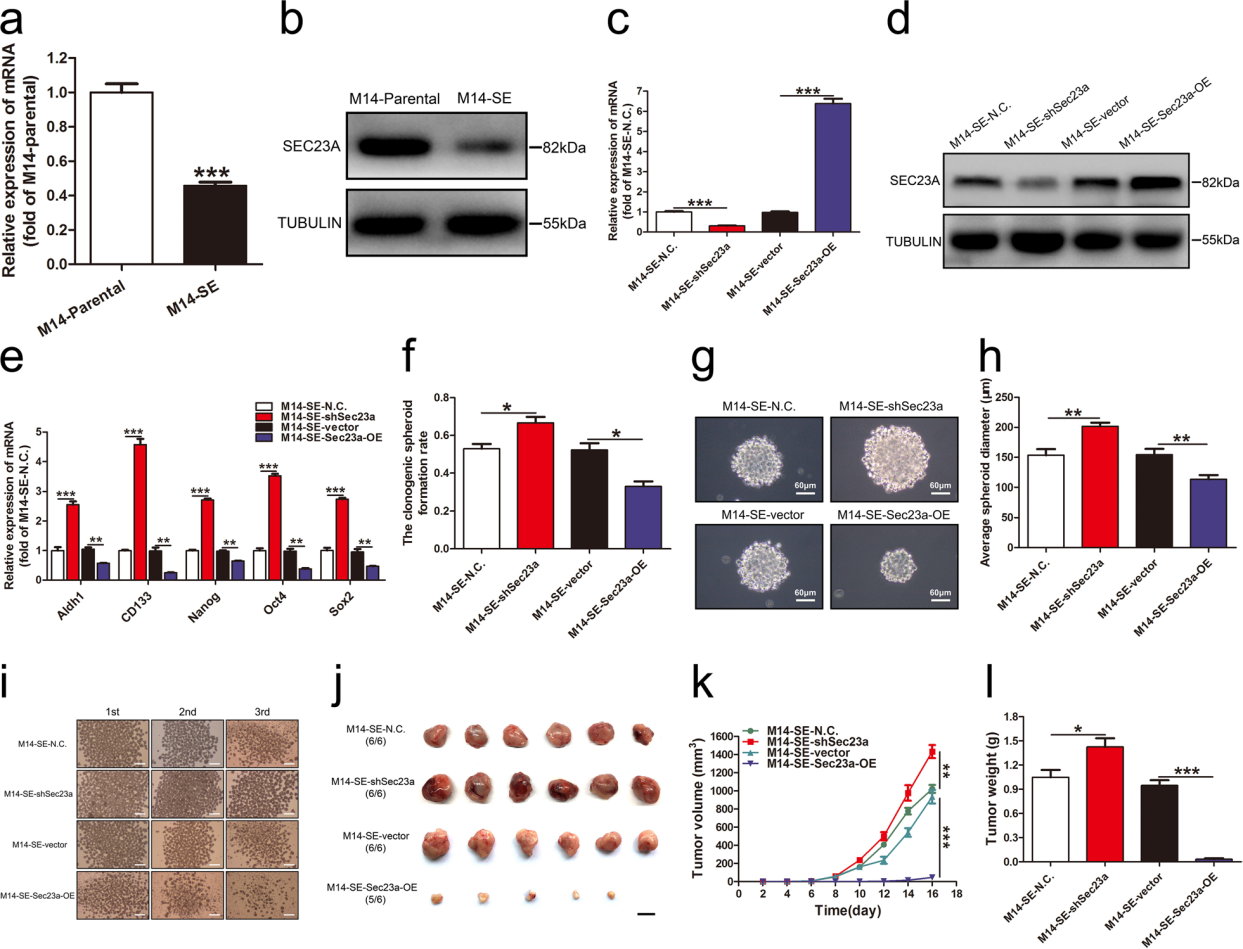
Sec23a inhibits the stemness of M14-SE cells. **a**, **b**. RT-qPCR and Western blot were performed to measure Sec23a expression in M14-Parental and M14-SE cells. **c**, **d** RT-qPCR and Western blot were performed to confirm effective gene manipulation of Sec23a expression in M14-SE cells. **e** Analysis of mRNA expression of Aldh1, CD133, Nanog, Oct4 and Sox2 in M14-SE cells with Sec23a interference or overexpression. GAPDH expression was used as a reference control. **f**–**h** Clonogenic spheroid formation rate, morphology and average spheroid diameter of M14-SE cells with Sec23a interference or overexpression in the single-cell cloning assay. **i** Serial spheroid formation assay of M14-SE cells with Sec23a interference or overexpression, bar = 200 µm. **j** Tumors resected from nude mice inoculated with M14-SE cells with Sec23a interference or overexpression, bar = 1 cm. (k and l). Tumor volume and weight, *P < 0.05, **P < 0.01, ***P < 0.001

The expression level of Sec23a in M14-SE cells was significantly lower than that in M14-Parental cells (Fig. 2a, b). To investigate whether Sec23a influences the stemness of M14-SE cells, the stemness features were analyzed upon alteration of Sec23a expression. Stable Sec23a interference or overexpression was achieved by lentivirus infection, and confirmed by RT-qPCR (Fig. 2c) and Western blot (Fig. 2d), respectively. RT-qPCR analysis clearly showed that Sec23a expression is negatively correlated with the expression of a set of stemness-related genes (Aldh1, CD133, Nanog, Oct4 and Sox2) in M14-SE cells compared with the controls (Fig. 2e). In vitro, the single-cell cloning assay and the serial spheroid formation assay showed Sec23a interference significantly enhanced the self-renewal of M14-SE cells while Sec23a overexpression weakened it inversely (Fig. 2f–i). In vivo, subcutaneous tumor transplantation assay was performed in BALB/c nude mice (Methods). Tumors derived from M14-SE-shSec23a cells were significantly bigger and heavier than those of M14-SE-N.C. cells. On the contrary, tumors derived from M14-SE-Sec23a-OE cells were significantly smaller and lighter than those of M14-SE-vector cells (Fig. 2j–l). These results collectively show that Sec23a inhibits the stemness of M14-SE cells both in vitro and in vivo.

### Autophagy promotes the stemness of M14-SE cells

**Fig. 3:**
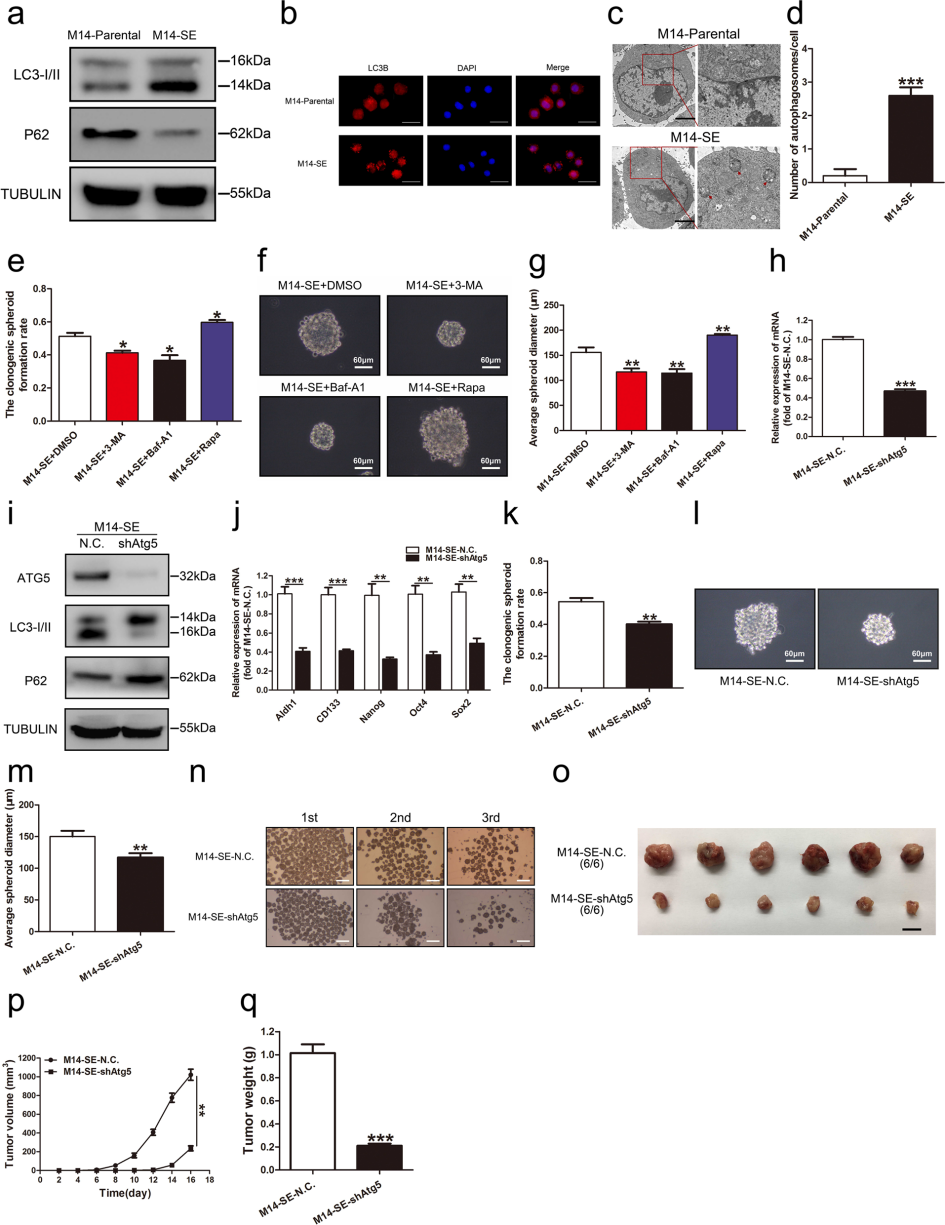
Autophagy promotes the stemness of M14-SE cells. **a** Western blot analysis for LC3I/II and P62 in M14-Parental and M14-SE cells. **b** M14-Parental and M14-SE cells expressed mRFP-LC3B fusion protein via adenovirus infection. Representatives of LC3B-positive puncta images were shown, bar = 20 μm. **c** Transmission electron microscopy of M14-Parental and M14-SE cells. Autolysosomes indicated by arrowheads, bar = 5 μm. **d** Mean number of detectable autolysosomes in each tumor cell, counted on transmission electron microscopy images. **e**–**g** Clonogenic spheroid formation rate, morphology and average spheroid diameter of M14-SE cells treated with DMSO, 3-MA, Baf-A1 and Rapa in the single-cell cloning assay. **h** and **i** RT-qPCR and Western blot analysis to confirm effective Atg5 interference in M14-SE cells. **j** Analysis of mRNA expression of Aldh1, CD133, Nanog, Oct4 and Sox2 in M14-SE cells upon Atg5 interference. **k**–**m** Clonogenic spheroid formation rate, morphology and average spheroid diameter of M14-SE cells with Atg5 interference in the single-cell cloning assay. **n** Serial spheroid formation assay of M14-SE cells with Atg5 interference, bar = 200 µm. **o** Tumors resected from nude mice inoculated with M14-SE cells with Atg5 interference, bar = 1 cm. **p** and **q** Tumor volume and weight, *P < 0.05, **P < 0.01, ***P < 0.001

While our prior work has shown for the first time the link between Sec23a and autophagy in regulation of M14 metastatic colonization [27], others have reported regulation of CSC stemness by autophagy [29, 30]. These observations prompted us to test whether autophagic activities differ in M14-parental and M14-SE. We thus compared the autophagic activity in the paired cell lines by: (1) the protein level of LC3 and P62 by western blot (WB), (2) mRFP-LC3B punta by immunofluorescence (IF), and (3) cytoplasmic accumulation of autolysosomes by transmission electron microscopy (TEM) analysis (Methods). We observed that autophagic activity was increased in M14-SE CSC compared to that of M14-parental, as determined by the three assays (Fig. 3a–d). Furthermore, the level of autophagic activity is positively related to the stemness of M14-SE cells, measured by the single-cell cloning assay. Whereas autophagy inhibitors (3-MA and Baf-A1) treatment weakened the stemness, autophagy activator treatment augmented it (Fig. 3e–g). These observations suggest that autophagy augments the stemness of M14-SE cells.

To confirm this finding, autophagy was inhibited by stable Atg5 interference (Fig. 3h, i) and the efficiency was verified by RT-qPCR (Fig. 3j). In vitro, Atg5 interference significantly weakened the self-renewal of M14-SE cells as determined by the single-cell cloning assay and the serial spheroid formation assay (Fig. 3k–n). In vivo, subcutaneous tumor transplantation assay in BALB/c nude mice showed that tumors derived from M14-SE-shAtg5 cells were significantly smaller and lighter than those of M14-SE-N.C. cells (Fig. 3o–q). Based on these observations, we concluded that autophagy augments the stemness of M14-SE CSCs both in vitro and in vivo.

### *Sec23a inhibits the stemness of M14-SE cells *via* inhibition of autophagy*

**Fig. 4:**
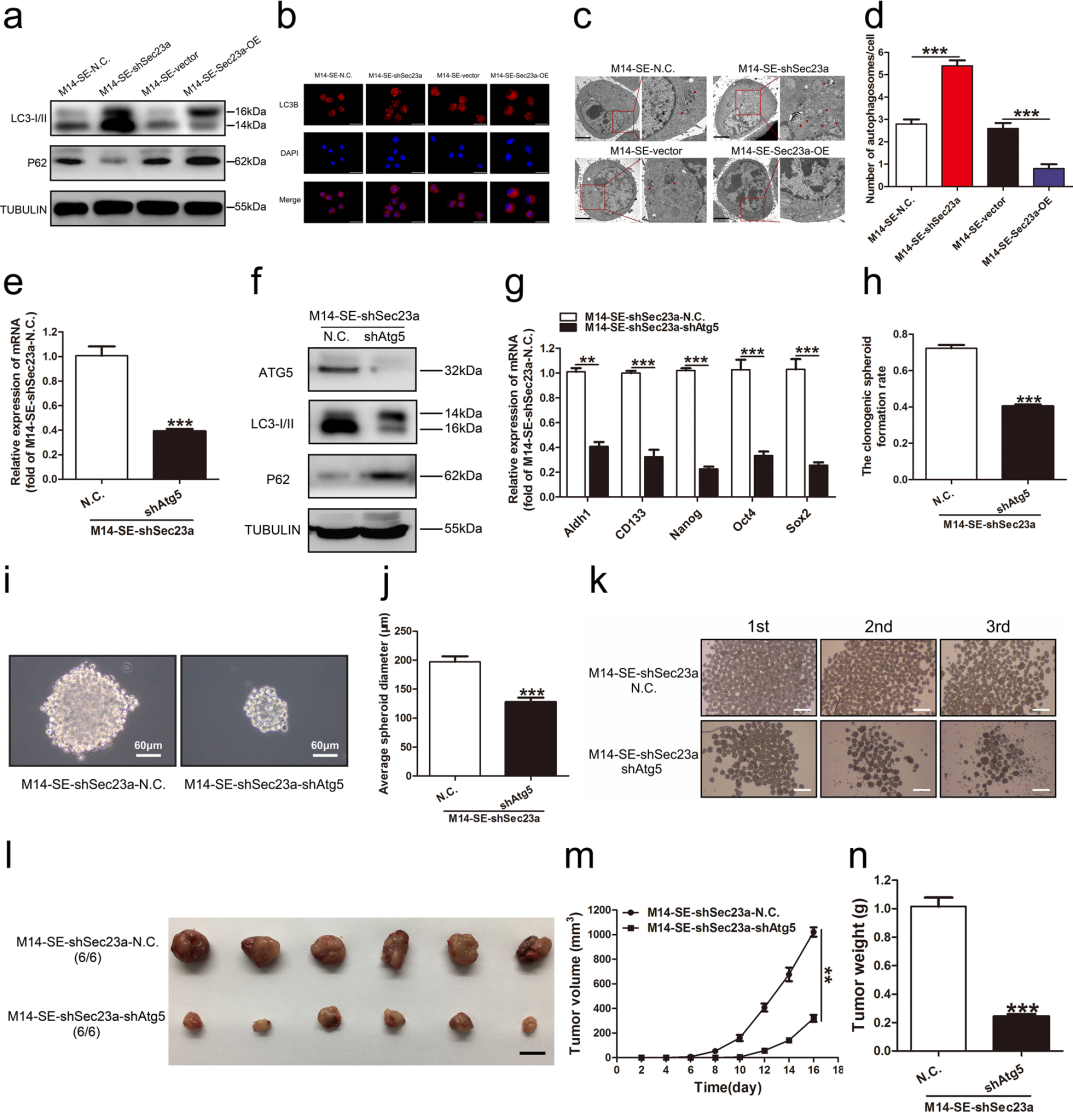
Sec23a inhibits the stemness of M14-SE cells via inhibition of autophagy. **a** Western blot analysis for LC3I/II and P62 in M14-SE cells with Sec23a interference or overexpression. **b** Representatives of LC3B-positive puncta images were shown in M14-SE cells with Sec23a interference or overexpression, bar = 20 μm. **c** Transmission electron microscopy of M14-SE cells with Sec23a interference or overexpression. Autolysosomes indicated by arrowheads, bar = 5 μm. **d** Mean number of detectable autolysosomes in each tumor cell, counted on transmission electron microscopy images. **e** and **f** RT-qPCR and Western blot analysis to confirm effective Atg5 interference in M14-SE-shSec23a cells. **g** Analysis of mRNA expression of Aldh1, CD133, Nanog, Oct4 and Sox2 in M14-SE-shSec23a cells with Atg5 interference. **h**–**j**. Clonogenic spheroid formation rate, morphology and average spheroid diameter of M14-SE-shSec23a cells with Atg5 interference in the single-cell cloning assay. **k** Sserial spheroid formation assay of M14-SE-shSec23a cells with Atg5 interference, bar = 200 µm. **l** Tumors resected from nude mice inoculated with M14-SE-shSec23a cells with Atg5 interference, bar = 1 cm. (m and n). Tumor volume and weight, **P < 0.01, ***P < 0.001

Thus far, we observed and confirmed that Sec23a inhibited the stemness of M14-SE cells (Fig. 2) and autophagy promoted the stemness of M14-SE cells (Fig. 3).Next, we evaluated whether Sec23a regulation of the stemness of M14-SE cells is dependent on autophagy using M14-SE-shSec23a and M14-SE-Sec23a-OE cell lines. WB analysis revealed a negative relationship between Sec23a expression and autophagy., i.e., autophagy was enhanced upon Sec23a interference and impaired with Sec23a overexpression in M14-SE cells (Fig. 4a). The inhibitory effect of Sec23a on autophagy was confirmed by mRFP-LC3B punta analysis (Fig. 4b). The formation of autolysosomes was analyzed by TEM. Autolysosomes were markedly increased in M14-SE cells with Sec23a interference and were decreased in M14-SE cells with Sec23a overexpression (Fig. 4c, d). These results confirm that Sec23a inhibits autophagy in M14-SE cells.

We next examined the molecular relationship between autophagy and Sec23a. We inhibited autophagy by stable Atg5 interference in M14-SE-shSec23a cells (Fig. 4e, f) in which stemness of M14-SE cells was augmented (Fig. 2). Atg5 interference inhibited stemness-related gene expression in M14-SE-shSec23a-shAtg5 cells (Fig. 4g). This observation was confirmed by in vitro single-cell cloning assay and the serial spheroid formation assay (Fig. 4h–k). In vivo, tumors derived from M14-SE-shSec23a-shAtg5 cells were significantly smaller and the mice were lighter than those from M14-SE-shSec23a-N.C. cells (Fig. 4l–n). These observations show convincingly that Sec23a inhibits the stemness of M14-SE cells by inhibition of autophagy.

### *Sec23a inhibits autophagy in M14-SE cells *via* inhibition of ER stress*

**Fig. 5:**
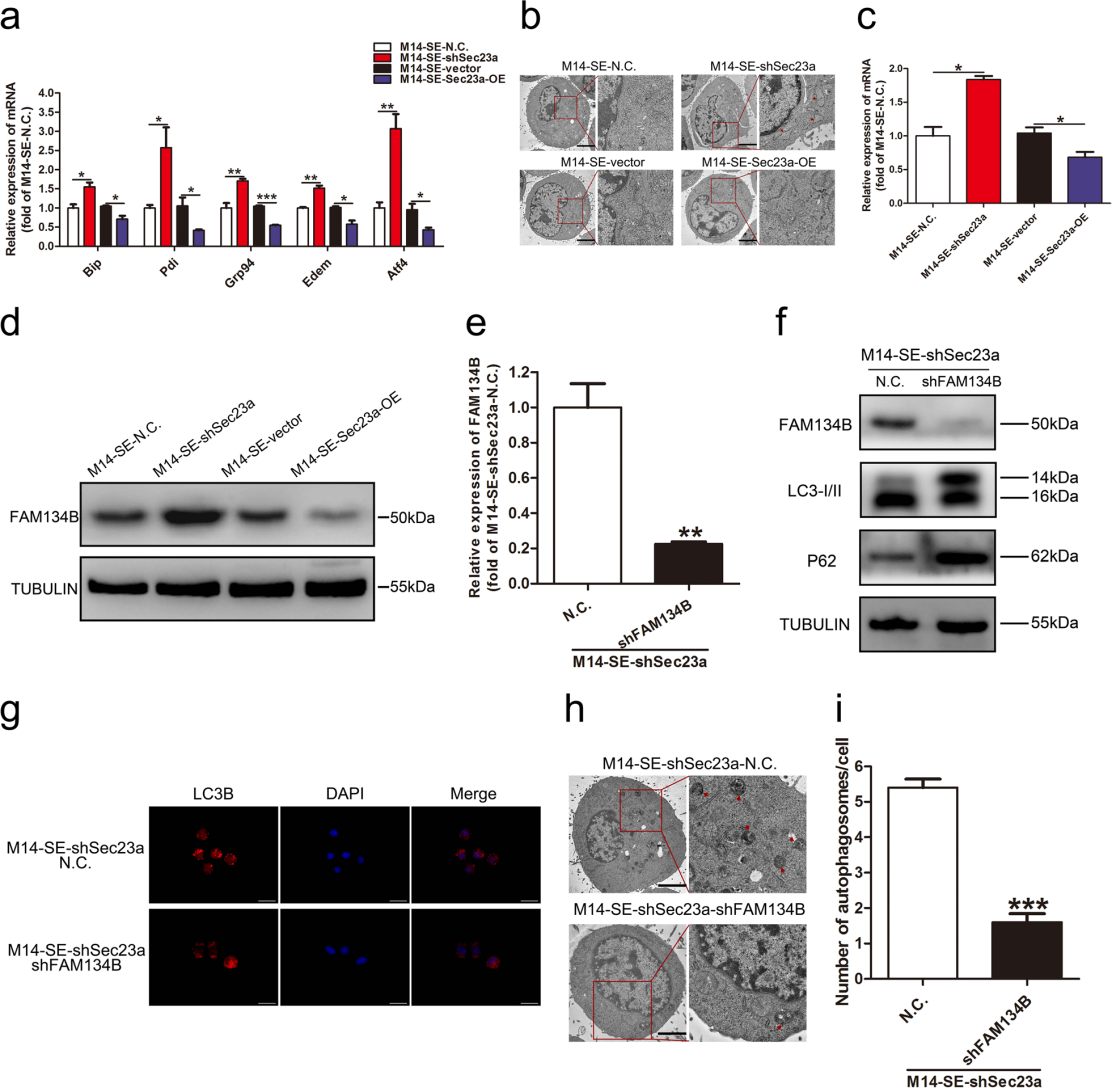
Sec23a inhibits autophagy in M14-SE cells via inhibition of ER stress. **a** Analysis of mRNA expression of ER stress marker genes in M14-SE cells with Sec23a interference or overexpression. **b** The morphology of ER in M14-SE cells with Sec23a interference or overexpression under transmission electron microscopy, bar = 5 μm. **c** and **d** RT-qPCR and Western blot were performed to measure FAM134B expression in M14-SE cells with Sec23a interference or overexpression. **e** RT-qPCR was performed to confirm effective FAM134B interference in M14-SE-shSec23a cells. **f** Western blot analysis for FAM134B, LC3I/II and P62 in M14-SE-shSec23a cells upon FAM134B interference. **g** Representatives of LC3B-positive puncta images were shown in M14-SE-shSec23a cells upon FAM134B interference, bar = 20 μm. **h** Transmission electron microscopy of M14-SE-shSec23a cells with FAM134B interference. Autolysosomes indicated by arrowheads, bar = 5 μm. **i** Mean number of detectable autolysosomes in each tumor cell, counted on transmission electron microscopy images. *P < 0.05, **P < 0.01, ***P < 0.001

Sec23a is responsible for the transport of secretory proteins from ER to Golgi apparatus. It was reported that Sec23a could regulate ER stress [22, 31]. To investigate the specific mechanism by which Sec23a inhibits autophagy, we first measured the level of the ER stress in M14-SE cells with Sec23a interference (M14-SE-shSec23a) or overexpression (M14-SE-Sec23a-OE) by assessing the expression of five ER stress maker genes (Fig. 5a). Sec23a expression was inversely related to the expression of ER stress genes (Fig. 5a), i.e., Sec23a appeared to inhibit ER stress. TEM showed the ER in M14-SE-shSec23a cells presented a typical stress response (Fig. 5b), consistent with the molecular findings (Fig. 5a).

Since ER stress can induce ER-phagy, a specific form of autophagy [32, 33], we next determined whether inhibition of ER-stress by Sec23a leads to reduction of ER-phagy, We found that the expression of FAM134B, an important ER-phagy receptor protein, was inversely related to Sec23a expression level in M14-SE cells (Fig. 5c, d). To examine whether FAM134B mediates Sec23a inhibition of ER-phagy, stable FAM134B interference was achieved by lentivirus infection in M140SE-shSec23a cells (Fig. 5e, f). WB analysis of P62 and LC3 lipidation showed that FAM134B interference significantly inhibited autophagy activity (Fig. 5f). The effect of FAM134B on autophagic activity was verified by mRFP-LC3B punta and autolysosomes formation (Fig. 5g–i). This set of results show that elevation of ER stress upon Sec23a interference, sensed by FAM134B will lead to augmented autophagic/ER-phagic activity.

### FAM134B promotes the stemness of M14-SE cells

**Fig. 6:**
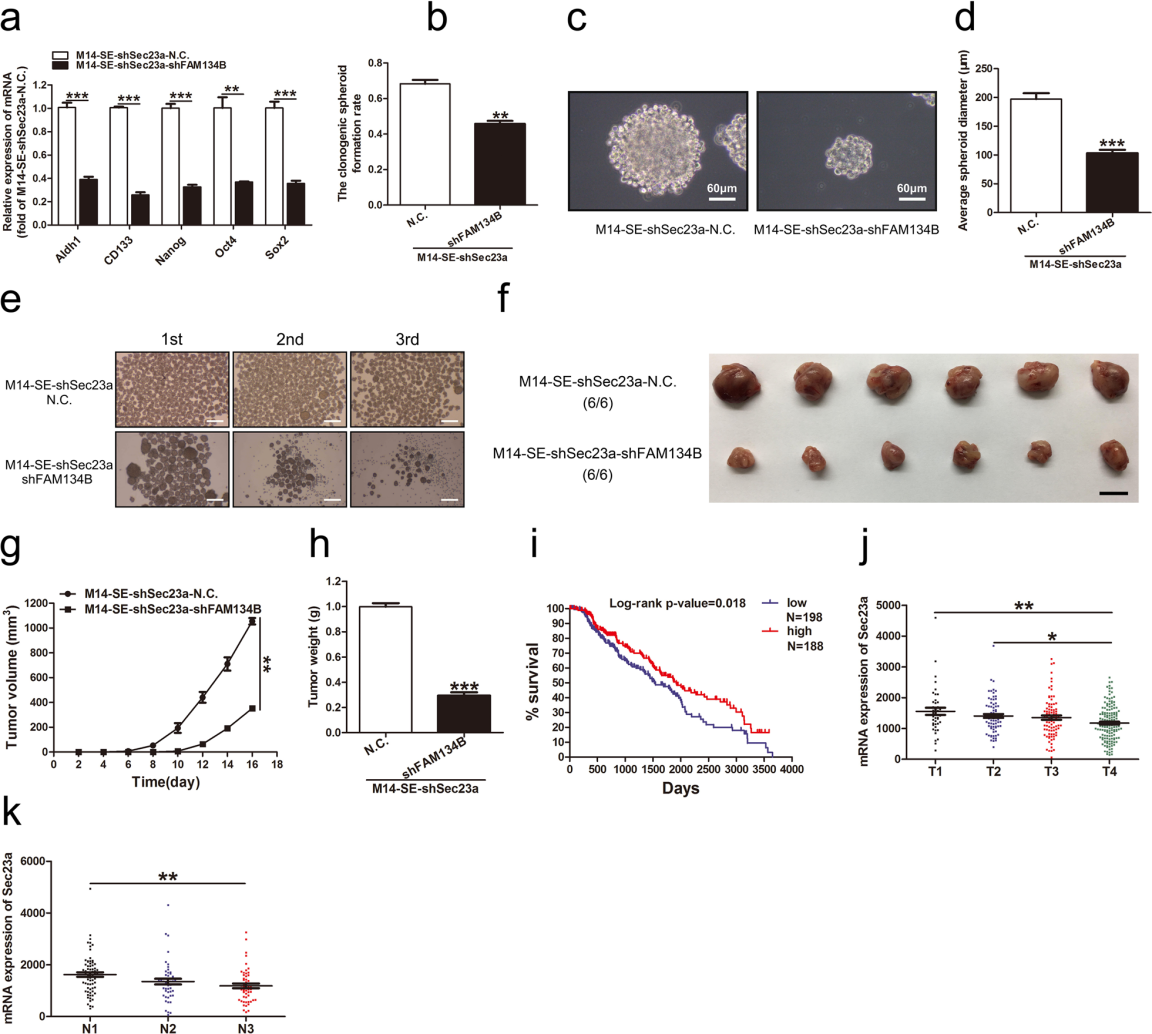
FAM134B promotes the stemness of M14-SE cells and Sec23a is a favorable diagnostic and prognostic marker for SKCM. **a** Analysis of mRNA expression of Aldh1, CD133, Nanog, Oct4 and Sox2 in M14-SE-shSec23a cells with FAM134B interference. **b**–**d** Clonogenic spheroid formation rate, morphology and average spheroid diameter of M14-SE-shSec23a cells with FAM134B interference in the single-cell cloning assay. **e** Serial spheroid formation assay of M14-SE-shSec23a cells with FAM134B interference, bar = 200 µm. **f**. Tumors resected from nude mice inoculated with M14-SE-shSec23a cells with FAM134B interference, bar = 1 cm. **g** and **h** Tumor volume and weight. **i** Low Sec23a expression levels indicated poor overall survivals for SKCM patients using Kaplan–Meier Plots analysis. **j** and **k** Correlation between Sec23a expression and tumor stages in SKCM patients using the TCGA database. *P < 0.05, **P < 0.01, ***P < 0.001

To confirm that ER stress/ER-phagy regulates the stemness of M14-SE cells, we assayed the expressions of stemness related genes upon FAM134B interference. FAM134B interference significantly inhibited the expression of stemness-related genes in M14-SE-shSec23a cells (Fig. 6a). In vitro, the single-cell cloning assay and the serial spheroid formation assay showed that FAM134B interference significantly hindered the self-renewal capability of M14-SE-shSec23a cells (Fig. 6b–e). In vivo, tumors derived from M14-SE-shSec23a-N.C. cells were significantly bigger and heavier than those of M14-SE-shSec23a-shFAM134B cells (Fig. 6f–h).

### Sec23a is a favorable diagnostic and prognostic marker for SKCM

To assess the clinical relevance of our novel finding that Sec23a inhibits the self-renewal of melanoma CSCs, we performed clinical data analysis using the TCGA (The Cancer Genome Atlas). Kaplan–Meier plots analysis revealed that skin cutaneous melanoma (SKCM) patients with high Sec23a expression levels had significantly better overall survivals in comparison with patients with low Sec23a expression level (Fig. 6i). Furthermore, Sec23a expression level was significantly lower in SKCM patients with advanced primary tumors and regional lymph node metastasis in comparison with SKCM patients of early stages (Fig. 6j, k).

### Sec23a inhibits the stemness of A375-SE cells via inactivation of ER stress and autophagy

**Fig. 7:**
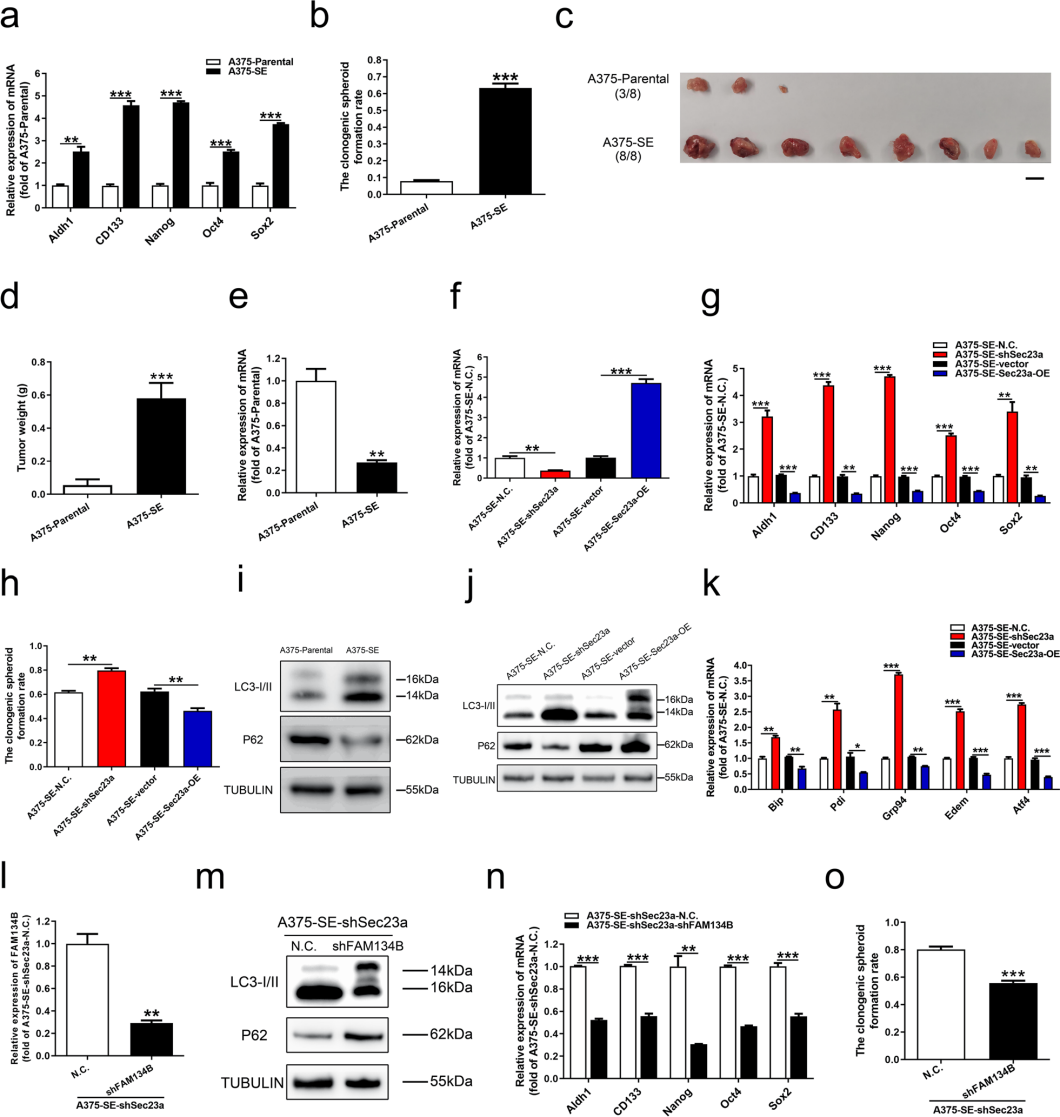
Sec23a inhibits the stemness of A375-SE cells via inactivation of ER stress and autophagy. **a** Analysis of mRNA expression of Aldh1, CD133, Nanog, Oct4 and Sox2. GAPDH expression was used as a reference control. **b** Assessment of the clonogenic spheroid formation rate of A375-Parental and A375-SE cells measured by the single-cell cloning assay. **c** Tumors resected from nude mice inoculated with A375-Parental and A375-SE cells, respectively, bar = 1 cm. **d** Tumor weight of nude mice to which A375-Parental and A375-SE cells were injected respectively. **e** RT-qPCR was performed to measure Sec23a expression in A375-Parental and A375-SE cells. **f** RT-qPCR was performed to confirm gene manipulation of Sec23a expression in A375-SE cells. **g** Analysis of mRNA expression of Aldh1, CD133, Nanog, Oct4 and Sox2 in A375-SE cells with Sec23a interference or overexpression. GAPDH expression was used as a reference control. **h** Assessment of the clonogenic spheroid formation rate of A375-SE cells with Sec23a interference or overexpression by the single-cell cloning assay. **i** Western blot analysis for LC3I/II and P62 in A375-Parental and A375-SE cells. **j** Western blot analysis for LC3I/II and P62 in A375-SE cells with Sec23a interference or overexpression. **k** Analysis of mRNA expression of ER stress marker genes in A375-SE cells with Sec23a interference or overexpression. **l** RT-qPCR was performed to confirm FAM134B interference in A375-SE-shSec23a cells. **m** Western blot analysis for LC3I/II and P62 in A375-SE-shSec23a cells with FAM134B interference. **n** Analysis of mRNA expression of Aldh1, CD133, Nanog, Oct4 and Sox2 in A375-SE-shSec23a cells with FAM134B interference. **o** Assessment of the clonogenic spheroid formation rate of A375-SE-shSec23a cells with FAM134B interference by the single-cell cloning assay. *P < 0.05, **P < 0.01, ***P < 0.001

To further verify the effect of Sec23a on the self-renewal of melanoma CSCs and the underlying mechanism is not limited to M14 and can be generalizable to melanoma, another melanoma CSC cell line named A375-SE was established from human melanoma cell line A375 via the same method as M14-SE. The expressions of stemness markers including Aldh1, CD133, Nanog, Oct4 and Sox2 in A375-SE cells were significantly improved in comparison with that in A375-Parental cells (Fig. 7a). Single-cell cloning assay and subcutaneous tumor formation assay were conducted to testify the enhanced stemness properties in A375-SE cells in vitro and *in vivo* (Fig. 7b–d). Unsurprisingly, the expression level of Sec23a in A375-SE cells was significantly lower than that in A375-Parental cells (Fig. 7e). Then stable Sec23a interference or overexpression was performed to investigate the effect of Sec23a on the self-renewal of A375-SE cells (Fig. 7f). RT-qPCR analysis showed that Sec23a significantly weakened the expression of stemness-related genes, including Aldh1, CD133, Nanog, Oct4 and Sox2 (Fig. 7g). And the single-cell cloning assay also showed Sec23a inhibited the self-renewal of A375-SE cells (Fig. 7h). Then the underlying mechanism of the inhibitory effect of Sec23a on the self-renewal of A375-SE cells was further investigated. The protein level of LC3 and P62 showed that autophagic activity was improved in A375-SE cells in comparison with A375-parental cells (Fig. 7i). Furthermore, the autophagic activity in A375-SE cells is negatively correlated with Sec23a expression (Fig. 7j). Moreover, Sec23a expression level was also negatively correlated with the expressions of a set of ER stress-related genes in A375-SE cells (Fig. 7k). Since FAM134B played a crucial role in the connection between ER stress and autophagy, stable FAM134B interference was achieved by lentivirus infection in A375-SE-shSec23a cells (Fig. 7l). WB analysis of P62 and LC3 lipidation showed that FAM134B interference significantly inhibited the autophagic activity in A375-SE cells (Fig. 7m). As expected, FAM134B interference observably restrained the expressions of stemness-related genes and the clonogenic spheroid formation rate of A375-SE cells (Fig. 7n, o). Collectively, these observations in A375-SE are comparable and consistent with those in M14-SE.

## Discussion

Human skin cutaneous melanoma (SKCM) is characterized by its high rate of metastasis and mortality [1]. More effective clinical management of SKMC requires improved understanding of the molecular mechanisms underlying melanoma stem cell self-renewal which may play a pivotal role in melanoma metastasis [34, 35]. Accumulating evidence have shown augmentation of CSC self-renewal by autophagy [36–38]. In a recent study, we reported for the first time that autophagy augments the stemness of lung CSCs by degrading ubiquitinated TP53. Further, Zeb1 is required for TP53 regulation of CSC self-renewal [13]. The present study has made a set of novel findings to elucidate a new CSC self-renewal mechanism that has not been described before, i.e., Sec23a inhibits the stemness of melanoma CSCs by inactivation of ER-stress induced ER-phagy.

Although a positive correlation between autophagic activities and the stemness of CSCs has been reported in breast, pancreatic, liver, osteosarcoma, ovarian and glioblastoma CSCs [39–42], the underlying mechanisms remain elusive. In the present study, we show that mechanistically, inhibition of Sec23a promotes ER stress and consequently FAM134B-conducted ER-phagy. Activation of autophagy by Endoplasmic reticulum (ER) stress is an evolutionarily conserved mechanism for the maintenance of cellular homeostasis [43, 44]. The ER stress-mediated autophagy is characterized by the generation of autophagosomes that include worn-out proteins, protein aggregates, and damaged organelles [45]. Signaling pathways of IRE1α, PERK, ATF6 and Ca2^+^ are necessary for the activation of ER stress-mediated autophagy [46–48], while the receptor-mediated ER-phagy that degrades the ER requires FAM134B [49–51]. FAM134B is an ER-resident receptor which contains a conserved putative LC3-interacting region (LIR motif). FAM134B binds to autophagy modifiers LC3 and GABARAP, which in turn facilitates ER degradation by ER-phagy [18]. We have revealed for the first time here that ER-phagy, activated upon sensing ER-stress by FAM134B, augments the stemness of melanoma CSCs.

Since the first report on the inhibitory role of Sec23a in cancer metastasis [24], mechanistic investigations have been focused on remodeling of tumor microenvironment by Sec23a-regulated cancer cell secretome at the site of distant metastasis [25, 52]. Consistent with the literature, we show that Sec23a inhibits metastatic colonization efficiency by changing the secretome to activate autophagic activity in melanoma cells. It is achieved via the Sec23a-S1008A-Beclin1 axis [27]. In a recent report, SEC23B, a homolog protein of the SEC23 family, can activate autophagy under nutrient deprivation condition [53]. However, secretome-independent role of Sec23a in regulation of tumor metastasis has not been reported. In addition, the connection between Sec23a and CSC self-renewal has not been established and reported prior to our present study. By employing the stable CSC cell lines derived from human melanoma cell lines M14 and A375, we show for the first time that Sec23a inhibits the self-renewal of melanoma CSCs via inactivation of ER-phagy. We have elucidated a novel CSC-based mechanism underlying the high metastatic rate of SKCM that has important clinical relevance. TCGA data mining and analysis show that Sec23a, the regulator of the ER-stress/FAM134B/ER-phagy axis, is a favorable diagnostic and prognostic marker for SKCM. Future studies using clinical cohorts are required to validate the diagnostic and prognostic importance of Sec23a in SKMC.

In summary, this study has elucidated a new mechanism underlying the regulation of autophagy on stemness, i.e. CSCs can exploit the SEC23A/ER-stress/FAM134B/ER-phagy axis for self-renewal. These findings provide new ideas for the exploration of the regulatory network of CSC self-renewal to develop CSCs-based therapy strategies for malignant tumors.

## Conclusions

In conclusion, our research findings reveal for the first time that Sec23a inhibits the self-renewal of CSCs via inactivation of ER-phagy. Sec23a is responsible for the transportation of secreted proteins from rough endoplasmic reticulum to Golgi apparatus. Inhibition of Sec23a significantly increasees ER-stress and augments the expression of FAM134B, which in turn through enhancing ER-phagy promotes the self-renewal of CSCs. FAM134B plays a pivotal role in ER stress-induced ER-phagy. The present study reveals for the first time that FAM134B promotes the self-renewal of CSCs. These findings improve our understandings of self-renewal regulations of CSCs and provide potential targets for CSCs-based therapy strategies.

## Data Availability

Data sharing not applicable to this article as no datasets were generated or analyzed during the current study.

## Abbreviations

CSCs: Cancer stem cells
ER: Endoplasmic reticulum
Sec23a: Sec23 homolog A
FAM134B: RETREG1, reticulophagy regulator 1
COPII: Coat protein complex II
SKCM: Skin cutaneous melanoma
OE: Overexpression
TEM: Transmission electron microscopy
IF: Immunofluorescence
TCGA: The Cancer Genome Atlas
